# The Principles of Hearable Photoplethysmography Analysis and Applications in Physiological Monitoring–A Review

**DOI:** 10.3390/s23146484

**Published:** 2023-07-18

**Authors:** Khalida Azudin, Kok Beng Gan, Rosmina Jaafar, Mohd Hasni Ja’afar

**Affiliations:** 1 Department of Electrical, Electronic & Systems Engineering, Faculty of Engineering and Built Environment, Universiti Kebangsaan Malaysia, Bangi 43600, Selangor, Malaysia; p111466@siswa.ukm.edu.my (K.A.); rosmina@ukm.edu.my (R.J.); 2 Department of Community Health, Faculty of Medicine, UKM Medical Centre, Universiti Kebangsaan Malaysia, Cheras 56000, Kuala Lumpur, Malaysia; drmhasni@ukm.edu.my

**Keywords:** in-ear photoplethysmography, hearable, health monitoring, signal processing

## Abstract

Not long ago, hearables paved the way for biosensing, fitness, and healthcare monitoring. Smart earbuds today are not only producing sound but also monitoring vital signs. Reliable determination of cardiovascular and pulmonary system information can explore the use of hearables for physiological monitoring. Recent research shows that photoplethysmography (PPG) signals not only contain details on oxygen saturation level (SPO2) but also carry more physiological information including pulse rate, respiration rate, blood pressure, and arterial-related information. The analysis of the PPG signal from the ear has proven to be reliable and accurate in the research setting. (1) Background: The present integrative review explores the existing literature on an in-ear PPG signal and its application. This review aims to identify the current technology and usage of in-ear PPG and existing evidence on in-ear PPG in physiological monitoring. This review also analyzes in-ear (PPG) measurement configuration and principle, waveform characteristics, processing technology, and feature extraction characteristics. (2) Methods: We performed a comprehensive search to discover relevant in-ear PPG articles published until December 2022. The following electronic databases: Institute of Electrical and Electronics Engineers (IEEE), ScienceDirect, Scopus, Web of Science, and PubMed were utilized to conduct the studies addressing the evidence of in-ear PPG in physiological monitoring. (3) Results: Fourteen studies were identified but nine studies were finalized. Eight studies were on different principles and configurations of hearable PPG, and eight studies were on processing technology and feature extraction and its evidence in in-ear physiological monitoring. We also highlighted the limitations and challenges of using in-ear PPG in physiological monitoring. (4) Conclusions: The available evidence has revealed the future of in-ear PPG in physiological monitoring. We have also analyzed the potential limitation and challenges that in-ear PPG will face in processing the signal.

## 1. Introduction

A hearable is a wireless in-ear component that integrates with biological signal monitoring technology. This type of wearable is designed to be inserted inside the ears in the shape of earphones or earbuds. In 2022, the hearable devices market had a value of USD 22.36 billion, and it is expected to increase to USD 93.80 billion, with a compound annual growth rate (CAGR) of 17.4% by 2027 [1]. The hearable market is expected to increase due to the need for wearable devices designed for health and wellness tracking purposes. The surge in demand for health and fitness tracking and smartphones is a substantial contributing factor.

To date, hearable devices are primarily used for sound applications, such as noise cancellation, audio analysis, sound masking, and spatial hearing [2]. However, some devices in the market have been developed for fitness tracking such as Amazfit PowerBuds, Samsung Gear IconX, Bose SoundSPort Pulse, LifeBEAM Vi Sense, Jabra Elite Sport Earbuds, and Philips Actionfit SN503 [3]. These earbuds are capable of tracking fitness activity and health monitoring, including heart rate, number of steps, distance, calories, and maximum rate of oxygen consumption during exercise (VO2Max).

Several authors have recently integrated hearables with more complex biometric monitoring technology, including gyroscopes, accelerometers, motion sensors, electrocardiograms (ECG), electroencephalograms (EEG), and photoplethysmography (PPG) signals. For instance, a study on in-ear sensing technology began by examining the detection and prevention of heat stroke [4]. This study utilized ear temperature, sweat, and sodium ion concentration as biomarkers of heat stroke. Some authors have attempted to utilize in-ear EEG to record spontaneous electrical activities of the brain and to analyze brain electrical signals associated with fatigue, sleep, drowsiness, and epilepsy [5,6,7,8].

Several studies on in-ear sensing technology have also explored in-ear ECG for heart monitoring [9,10,11,12]. Zhang et al. developed a study on health monitoring to measure blood pressure and heart rate, utilizing machine learning to eliminate motion with a combination of Ear PPG and Ear ECG [11]. They established the feasibility of detecting the depolarization of the heart in the QRS wave of in-ear ECG with lower signal-to-noise ratios. In-ear pulse oximetry has been previously studied by a variety of groups to measure the oxygen saturation in the blood [12,13,14,15,16,17,18,19]. Various in-ear PPG studies have focused on observing and measuring other vital signs such as HRV, blood oxygen saturation (SPO2), and respiration rate (RR) [14,15,19,20,21]. These vital signs are extracted from raw PPG signals that are detected from various measurement sites using signal processing methods. Due to its simplicity and value-added features such as health monitoring in earbuds and wearables, PPG has gained a lot of interest from manufacturers and researchers. On the other hand, it is worth describing the principle of PPG in brief before conducting an in-depth review of in-ear PPG and its applications. 

**Figure 1:**
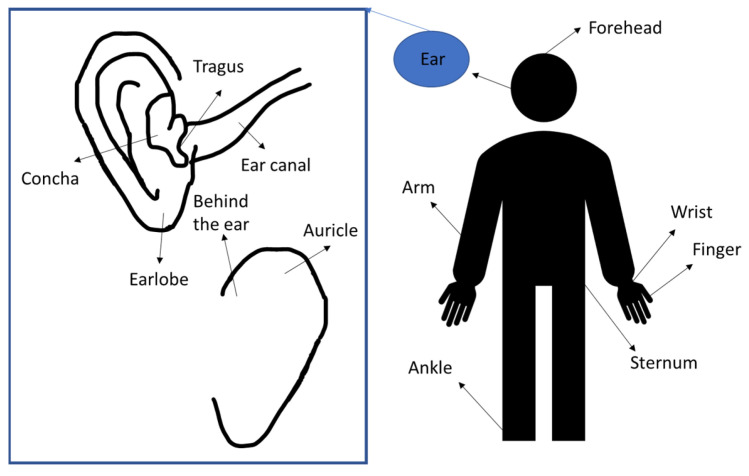
Common placement of PPG sensors.

PPG uses light to assess variations in blood circulation volume by measuring the light absorption on the tissue. The PPG system consists of a combination of photo emitter and photodetector. The PPG measurement area plays a pivotal role in PPG signal analysis. The waveform of the PPG signal is influenced by different anatomical regions, which are characterized by distinct tissue thickness, skin pigmentation, and density of blood vessels and bone structures. PPG signals are commonly measured at the wrist [22,23], finger, and earlobes. They can also be measured in other sites including forehead [15,24], upper and lower arm [25], abdominal area [26], and different ear areas (external ear cartilage, superior and inferior auricular region, and external auditory canal) as shown in Figure 1 [27,28].

While PPG devices are common in the clinical setting for measuring heart rate and blood oxygen saturation, the PPG sensor is usually positioned on the patient’s finger. Previous studies have observed PPG features related to the cardiovascular system that can observe changes in blood vessels such as systolic amplitude, pulse width, pulse area, and pulse interval [29,30,31,32,33,34]. Systolic amplitudes are correlated with systemic vascular resistance and cardiac output, which are reported to be useful in determining blood pressure. Pulse width is described to be correlated with microvascular expansion better than systolic amplitude [29,31,32,35]. Pulse interval is equivalent to a complete heart cycle that is associated with an R-R interval of a QRS interval in an ECG signal. A study on the prediction of diastolic and systolic blood pressure using PPG signal has been explored [36].

With the rapid development of earbud technology, health features become important in many wearables and hearable devices. A combination of personalised health information such as heart rate, oxygen saturation, body temperatures, and other vital signs gives users the ability to understand their own health condition while listening to music and exercising. To date, many studies have been previously conducted into the measurement of blood oxygen saturation from the ear [12,13,14,15,16,17,18,19]. Budidha and Kyriacou investigated SPO2 from ear canals in cold temperature conditions [16,37]. They established that in comparison to the finger PPG signal, the signal obtained from the ears is more accurate during hypothermia. Venema et al. engaged with an in-ear pulse oximetric sensor under laboratory settings and in an operation room during surgery [13,21]. In-ear SPO2 measurement under laboratory settings met the measurement specificity requirement for a pulse oximeter. However, under the clinical conditions, the requirement was not met [13]. Davies et al. performed non-invasive respiratory monitoring using in-ear PPG sensors [18,19]. They extracted a respiratory waveform from the in-ear PPG signal where the blood oxygen saturation (SPO2) and breathing rate were calculated, while chronic obstructive pulmonary disease (COPD) was estimated and classified. [18,19]. Measuring vital signs from the ear is of interest because of the limitations of the current measurement methods including motion error and reduced peripheral perfusion, particularly in life-threatening situations.

Various in-ear PPG studies have focused on observing and measuring other vital signs such as HRV, blood oxygen saturation (SPO2), and respiration rate (RR) [14,15,19,20,21]. Ferlini et al. and Venema et al. observed the ability of in-ear PPG sensors in measuring HR, HRV, SPO2, and RR [14,38]. Ferlini et al. explored the accuracy of PPG measurements at different PPG sensor positions around the ears with motion [14]. Venema et al. captured the in-ear PPG vital parameters to automatically estimate normal and irregular heart rhythm [38]. These studies established in-ear PPG as an alternative heart monitoring technique, especially to monitor heart rhythm. The method is affordable and non-invasive as it does not need skin preparation, patches, or wires for signal acquisition.

Previous studies have demonstrated the possibility of applying in-ear PPG technology for heart rate measurement [39,40]. Venema et al. proposed an in-ear PPG system for cardiorespiratory monitoring, while Pedrana et al. explored a hearable PPG system for continuous cardiac monitoring [38,40]. Existing research, including Vogel et al. [20], examined the measurement of heart rate (HR) from a PPG signal positioned inside the ear canal, while Poh et al. [12] placed the PPG sensors on the concha of the ear for heart monitoring where the HR and heart rate variability (HRV) were assessed.

Another study by He et al. presented a vital signs monitoring system at the ear using the combination of ear PPG signal with ECG and ballistocardiogram (BCG) to measure the pre-ejection period (PEP), stroke volume (SV), cardiac output (CO), and pulse transit time (PTT) [41]. He et al. explored that PTT can be measured by associating the ECG and PPG signals while PEP, SV, and CO measurements utilized BCG and ECG. The ear with its natural support was demonstrated to be a point for physiological signals measurement.

Cardiovascular diseases are still the main cause of death in Malaysia and globally. Continuous monitoring of the heart and cardiovascular condition of an individual has become important for the prevention and early detection of any potential heart problem, especially during physical activities, to avoid sudden cardiac death. PPG is among the technologies for the continuous monitoring of heart and cardiovascular conditions. As such, there is a number of research in hearable technology that has examined the potential of ear PPG for cardiovascular and respiratory monitoring. The ear consists of numerous micro vascularisations and major vascular that can reflect various physiological signals for these applications. Furthermore, earbuds provide a more convenient and comfortable alternative to finger PPG, which is currently being used in clinical settings. Additionally, the ear is less susceptible to motion artifacts compared to finger and wrist PPG, as the head is not as freely movable as the hand and finger. The positioning of the sensor inside the ear canal enables more accurate measurements of physiological signals than finger and wrist PPG. The location of the sensor in the ear can also reduce the interference of external ambient light and temperature changes.

Given the importance and growth of interest in in-ear PPG, this review explores the in-ear PPG technology and its applications through the previous literature and research. This study will examine the current technology of in-ear PPG including its measurement configuration and underlying principle, waveform properties, processing technology techniques, and characteristics extraction. This study is anticipated to have a positive impact on the application of in-ear PPG, which is increasingly in demand with the recent surge of mobile healthcare for healthcare monitoring.

The key contributions of this article are listed in the following in order:

1. This paper provides an overview of the current technology of the in-ear PPG including its principle, system, processing technology, and its applications in clinical and physiological monitoring.
2. This paper compiles published articles related to in-ear PPG technology and applications. It provides a bibliographic analysis of a few selected papers and reviews their principle, processing techniques, and clinical monitoring features applications.
3. This paper highlights in-ear PPG processing and applications in physiological monitoring topics. This review will provide a holistic analysis of a few selected papers and can be served as a foundation for further implementation in in-ear PPG research.

This paper is embodied in the following order. In Section 2, we present the approaches we used in achieving this review. In Section 3, we provide an overview of the in-ear PPG including its principle, system, processing technology, and applications in physiological monitoring. In Section 4, we discuss the limitations and the gaps in the in-ear PPG problems. In Section 5, we provide the concluding remark of the paper.

## 2. Materials and Methods

### 2.1. Search Structure

**Figure 2:**
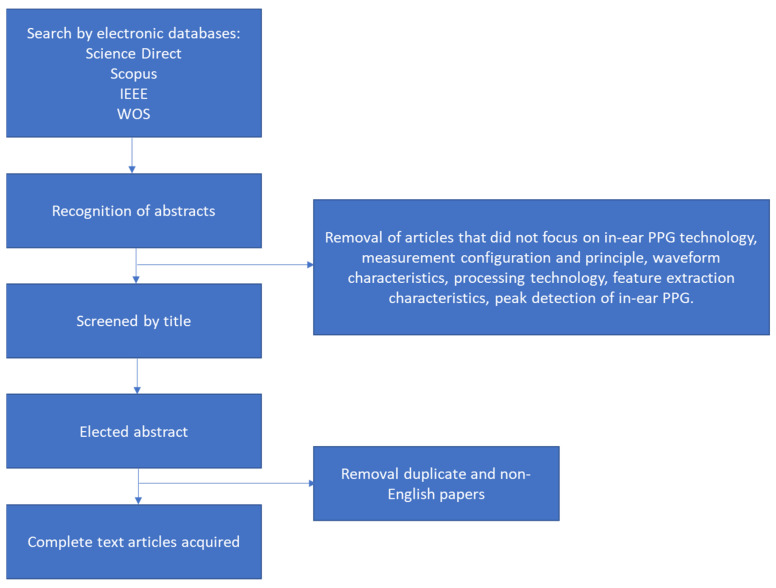
Search strategy.

A literature search for reference was conducted to study in-ear PPG signal processing and its applications in cardiovascular and respiration monitoring. A review of the literature was performed using the following databases: Institute of Electrical and Electronics Engineers (IEEE, Piscataway, NJ, USA), ScienceDirect (Elsevier, Amsterdam, The Netherlands), Scopus (Elsevier, Amsterdam, The Netherlands), and Web of Science (Clarivate, London, UK) to discover appropriate articles published until December 2022. Related articles were determined by using the following terms and keywords: hearable, in-ear photoplethysmography, ear canal, ear canal pulse oximetry, heart rate, SPO2, cardiovascular, and cardiac arrhythmia combined. The process of selection is done following the sequence in Figure 2. 

### 2.2. Inclusion Criteria

The main inclusion criteria for this review were limited to articles published in the English language with the main condition being that an article must focus on in-ear PPG technology, measurement configuration and principle, waveform characteristics, processing technology, feature extraction characteristics, and peak detection of in-ear PPG.

### 2.3. Review Process

The searched articles were filtered before being selected for review. In this process, any articles that did not meet the inclusion criteria based on their title and abstracts were eliminated. The remaining articles were reviewed by authors and were grouped according to the characteristics and processing procedure of in-ear PPG.

## 3. Results

### 3.1. In-Ear PPG Principle

The ear is a hearing sensory organ constituted of three regions: the outer ear (pinna), the middle ear (concha), and the inner ear (auditory canal). The ear is suggested to be a convenient location for a wearable because of its generally accepted area by consumers for communication devices such as earphones and earbuds. For the PPG signal, the ear position is nearer proximity to the heart as opposed to the wrist and finger. The ear PPG has more benefits compared to finger probes used for pulse oximetry. The ear is possible for PPG detection because of several major blood vessels supplying blood including the superficial temporal artery, superior auricular artery, and external carotid artery. A high concentration of blood vessels with multiple capillaries can be an alternative for a PPG sensor placement.

A previous study has established that the PPG signal from the ears is more accurate than finger PPG during hypothermia, hypovolemia, or sepsis. The PPG signal from the ear does not suffer from a reduced peripheral perfusion [42]. The ear canals are believed to be unaffected by the cardiovascular centralization [42]. The ears are shown to maintain the internal blood volume and do not adapt to temperature changes compared to peripheral areas where they will experience vasoconstriction, which is the restriction of blood flow that occurs in a cold environment [16]. The structure of the ear that is surrounded by skin maintains the internal blood flow level.

It has demonstrated that in-ear PPG has a faster response to blood oxygen changes compared to finger PPG and it can accurately detect hypoxia and sleep apnea [19,21,38]. Research has shown that the ear PPG signal is more susceptible to a variation in intensity arising from respiration.

### 3.2. In-Ear PPG Placement

An in-ear PPG system can be measured from the outer ear, which is conveniently accessible for an earpiece. Several locations of ear sensors have been identified in previous studies (see Table 1) which are as follows: at the earlobe, at the auricle area, in the ear canal, and at the tragus [16,18,38,39,40]. Previously published studies have always focused on studying PPG signals from the auditory. The anatomy and shape of the external auditory meatus could be a natural support for the sensor [40]. This could prevent the displacement of the sensor.

Budidha and Kyricou collected a PPG signal from inside of the ear with an ear crotchet that hangs on the brink of the helix. The sensor was positioned inside the auditory canal and maintains the probe at a fixed position and prevents any displacement to reduce motion artifacts. The ear canal has manifested to be less responsive to blood volume fluctuations during hypothermia and it has proven to be highly sensitive to intensity variations that occur due to respiration [16,18]. Venema et al. suggested collecting a PPG reflective signal from the tragus while wearing a measurement system behind the auricle [38,43]. Davies et al. observed a higher spectral power of pulse amplitude variations due to respiration and higher respiration-induced intensity variation power from the ear by 8.5 folds [18].

Ferlini et al. investigated three locations around the ears for the PPG sensor placement such as behind the auricle or pinna, in front of the auricle or concha, and in the ear canal [14]. In their useful analysis, Ferlini et al. concluded that the ear canal showed minimal errors and the slightest intersubjective variability across different movements (speaking, running, and walking) [14]. However, the PPG signal in the ear canal still produced motion artifacts even though its location is the most stable part of the body.

Pedrana et al. explored 18 different locations on the body such as fingers, wrist, forehead, ankle, ear, and sternum area where a good PPG signal quality could be obtained. The perfusion index was correlated with the quality of the PPG signal even though the perfusion index could vary between individuals. The perfusion index was found to be higher at fingers, ears, and nose compared to other sites, which indicates a good quality PPG signal at these location [40].

Vogel et al. compared two in-ear locations: near-to-tragus and away-from-tragus [20]. Positioning the PPG sensor deeper in the ear canal when walking produced higher AC amplitude resulting in a better SNR. This also increases the sensitivity to the artifacts caused by chewing and talking, which reduced the signal quality.

Tigges et al. constructed the prototype with optical sensors in two different orientations to the ear canal, which are axial and radial alignment [42]. The radially oriented sensor provided better signal quality as better light absorption between the sensor and the ear canal.

### 3.3. In-Ear PPG System

**Figure 3:**
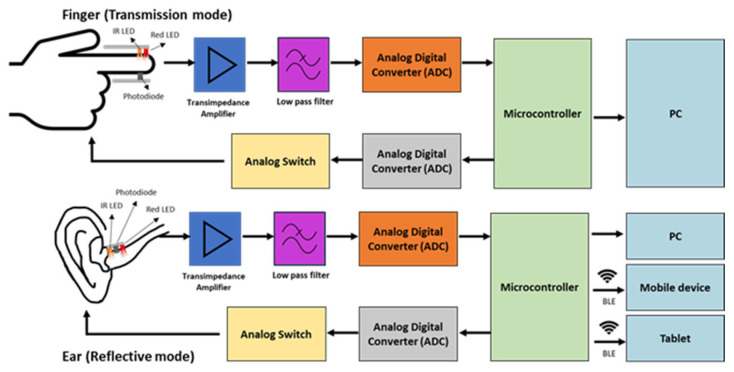
Difference of block diagram of in-ear PPG (**below**) and finger PPG (**above**).

There are several digital PPG sensors available that have been used in clinical studies to measure PPG pulse wave. All studies related to in-ear PPG were operating in reflectance mode. Reflectance mode mirrors the light on the skin, which is then captured by the photodetector located side by side. The basic of the block diagram of the in-ear PPG can be observed in Figure 3. 

Davies et al. and Pedrana et al. utilized MAX30101 (San Jose, CA, USA) by Maxim Integrated in their studies [18,19,40]. MAX30101 digital PPG sensor chip consists of three internal LED drivers with different wavelengths, green (537 nm), red (660 nm), and infrared (880 nm) for SPO2 and heart rate measurement, a photodiode to measure the reflected light, and low-noise amplifier with surrounding light rejection [18,19]. The MAX30101 has bypass capacitors to absorb the noise and level shifters that allow digital communication between 1.8 V and 3 V domains [18,19].

Pedrana et al. explored the relationship between signal quality and the LED, measurement tissues, motion artifacts, and probe contact force. The study determined that for a subject without motion, LED with a current of 10 mA (4% of duty cycle) had a good signal quality for the three colours of LED (green, red, and infrared) [40]. The red and infrared would begin saturating at 25 mA of intensity caused by the analog-to-digital converter. However, green would never saturate even at the maximum current of 50 mA [40]. The choice of LED colours relies on the measurement of tissue and area where longer wavelengths penetrated profoundly into the tissue. The infrared had a higher penetration depth between 0.8 mm and 1.5 mm compared to green, which penetrated at the maximum depth of 0.6 mm [40].

Budidha et al. used LEDs and a photodetector from Excelitas technologies (Waltham, MA, USA), from which the LEDs emit light at the infrared region (870 nm) and red region (658 nm). The maximum spectral sensitivity of the photodiode is at 900 nm (SR 10 BP-PG), of which the photodiode operating area is 0.65 mm^2^ [16]. The photodiode and LEDs were designed to capture PPG signals from the bottom surface of the auditory canal and both were located at a distance of 5 mm from each other [16]. Previous studies have described that a better signal-to-noise ratio can be achieved with the distance of the LED and the photodiode between 4 and 5 mm [44].

Vogel et al. developed an in-ear PPG system with a receiver diode, a monitor diode, and a detector diode positioned at one side of the sensor. An optical barrier made from the glass is placed between the receiver and detector diode to minimize the optical crosstalk [20]. An artifact recording system has been developed to measure the movement effects towards the PPG signal. The system consisted of a red and infrared in-ear PPG sensor, a 2D and 3D accelerometer that was fixed at the head and the hip to record walking and jaw motion, and a clinically approved reference system worn on the finger to validate the in-ear PPG signal [20].

In a study conducted by Tigges et al., they developed an in-ear sensor system with an OSRAM SFH 7051 optical sensor (Munich, Germany), a Texas Instruments AFE 4490 analog front-end (Dallas, TX, USA), and a STMicroelectronics STM32L476 microcontroller (Geneva, Switzerland). The optical sensor consisted of three green light LED (530 nm) and a photodetector. The analog front-end comprised an 8-bit current resolution LED driver and a 3-stage receive channel that conducted a sequential current to voltage conversion, AC signal amplification, removal of DC offset, and filtering for analog signal conditioning [42]. The microcontroller was connected through an SPI interface operating at 8 MHz, which allowed for the storage of all measurement and events logs on a memory card. An automated calibration was executed to achieve a maximum pulse amplitude that accommodates the analog signal conditioning [42].

Venema et al. demonstrated an in-ear PPG system connected via Bluetooth [38]. The PPG sensor is in reflectance mode. The photodiode and two LED (760 nm and 905 nm) in the device are connected anti-parallel with a separation of 3 mm and detached by an optical barrier to prevent light disturbance [21,38,43]. The red and infrared LED emitted a light intensity of 2.74 mW and 3.69 mW, respectively, with a current of 30 mA. The signal is converted using an Analog Digital Converter and pre-processed using the embedded microcontroller MSP430F1612 by Texas Instrument.

### 3.4. In-Ear PPG Signal Processing

**Figure 4:**
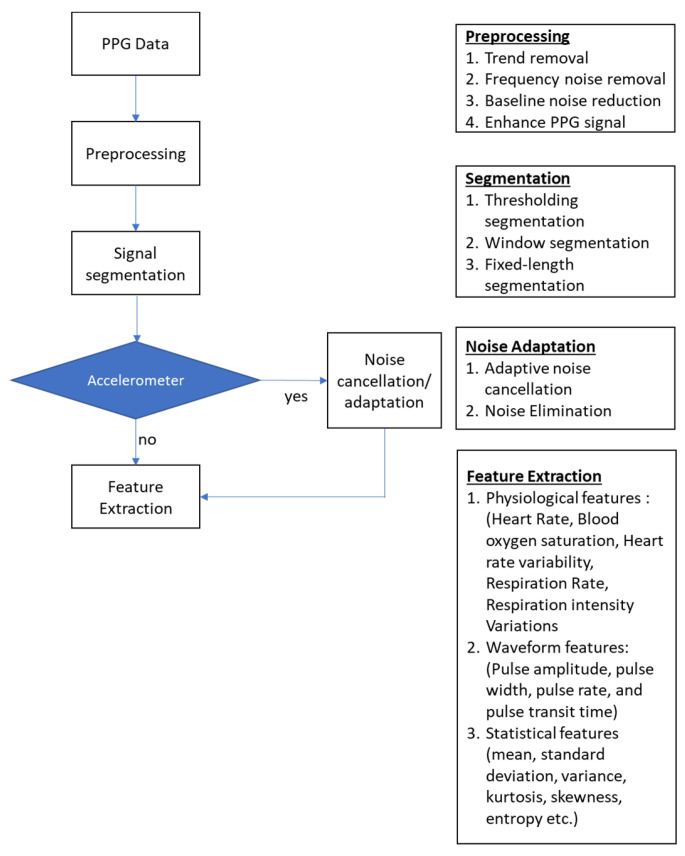
PPG signal processing block diagram.

The processing method for in-ear PPG waveform adheres to well-established principles (see Figure 4). After acquiring the raw in-ear PPG signal using a sensor positioned inside the ear, the signal is pre-processed to remove noise, baseline drifts and artifacts. The pre-processed signal is then segmented into individual pulses. The segmentation is implemented to extract features and information from the signal. Some of the sensors are coupled with accelerometer where noise cancellation and adaptation are applied to the signal. 

#### 3.4.1. Waveform Characteristics

The structure of PPG waveform acquired from ear canals are different from the PPG pulse obtained from the finger. A dicrotic notch from the ear PPG signals is almost invisible. The PPG amplitude of the in-ear PPG sensor was found to be lower compared to the finger PPG. Davies et al. have considered the following reasons for reduced amplitude, which include the less vascular density of the tissue in the auditory canal, poor contact between the sensor and the external auditory canal tissue, and the smaller amplitude of the carotid arterial pulse pressure in the ear canal [19]. However, the power of pulse amplitude variation was observed to be higher in the ear canal. This may be due to the auditory position, which is near the carotid artery [18].

The in-ear PPG waveform was classified into pulsatile and non-pulsatile components [14]. The non-pulsatile component is characterized by a fixed amount of light being absorbed by the tissues, bones, and muscles. The amount of light that is measured by the photodetector was reduced in intensity and is directly related to the non-pulsatile component, which is also referred to as DC component [14]. The pulsatile component is the modulated component of the PPG signal that is directly associated with the arterial blood volume variation, also known as the AC component [14].

Basic PPG features that are used clinically and are acquired directly from the in-ear PPG pulse wave are systolic amplitude. The PPG signal is usually detrended to remove any biased at 0 Hz. This detrending method is usually performed in the time domain. Pedrana et al. removed the trend of the PPG signal; a second-order Butterworth filter with a cut-off frequency of 0.5 Hz was applied to low-pass filter the signal, and the resulting filtered signal was then subtracted from the original PPG signal [40]. They processed the signal by combining the detrended PPG signal from the infrared LED and red LED. Davies et al. detrended a raw PPG signal to remove any drift and to ensure no bias at 0 Hz during the spectral power calculation [18].

#### 3.4.2. Signal Pre-Processing

Various pre-processing methods were previously used in processing in-ear PPG signals (see Table 2). The majority of the pre-processing methods for in-ear PPG signals involve the use of frequency filtering to eliminate low and high-frequency, similar to the pre-processing techniques used for standard PPG signals. Several authors isolated the DC component by applying a low-pass filter on the PPG signal. The cut-off frequency is varied among authors. Pedrana et al. and Venema et al. applied the second-order Butterworth low-pass filter with a cut-off frequency of 0.5 Hz [21,40]. Davies et al. applied the low-pass filter onto the raw signal at 0.01 Hz [19].

Venema et al. applied two Butterworth low-pass filters in processing the in-ear PPG. First, they applied a Butterworth fifth-order filter to separate the cardiac activity from an almost sinusoidal pulse wave with a cut-off frequency of 10 Hz equivalent to ten times the healthy peoples’ resting heart rate [21]. Then, a second low-pass filter to isolate the DC component [21]. Tigges applied two threshold values to observe the signal quality of the PPG. The upper limits were set at 10 Hz and 20 Hz, while the lower limit was set at 0.5 Hz. A Linear Finite Impulse Response (FIR) filter was performed for frequency filtering in estimating SPO2 during hypothermia [16]. Venema et al., in their exploration of in-ear PPG for heart rate monitoring during sleep, employed the fourth-order bandpass Butterworth filter with a set of cut-off frequencies at 0.8 and 6 Hz [43]. The 0.8 Hz for low-pass frequency was chosen to ensure an adequate gap from the respiratory frequency band. They applied a second-order Butterworth filter with cut-off frequencies of 0.1 and 0.33 Hz to study the respiration rate [43].

In obtaining the AC components within the in-ear PPG measurement, the lower boundary of the passband filter is usually determined between 0.5 Hz and 1 Hz [14,18,19]. In lower bound frequency filtering, the objective is to remove the respiratory component in the 0.1 to 0.5 Hz frequency band and eliminate the DC component that is normally situated below 0.1 Hz.

The upper bound of the passband was filtered between 4 Hz and 30 Hz [14,16,18,19]. Some authors set the upper bound of the passband to 4 and 4.5 Hz, considering the maximum heartbeat is at 240 beats per minute [20]. Venema chose 6 Hz to ensure that all frequencies of the original PPG pulse wave morphology were included [43]. Some authors fixed the upper frequency at 10 Hz, which, according to them, is when the heart beats at a rate of 150 beats per minute (2.5 Hz); the minimum frequency of the fourth harmonics is at 10 Hz [14,16,37]. The fourth harmonics in the frequency domain contain all of the major frequency components of PPG signals. While some authors have utilized an upper bound range up to 30 Hz, the frequency range up to 20 Hz has been found to contain important information about artery compliance, arterial blood pressure, and peripheral vascular compliance [18,19,46,47].

A moving average filter and a moving difference filter appeared to be implemented in pre-processing the signal to smoothen the signal and enhance the peak. A moving average filter was employed to smooth the AC and DC component [21]. A 20-s window, which contained a complete cycle of a pulse wave was used to compute the R-values [21]. A moving difference filter was implemented by Pedrana et al. to estimate the heart rate. They estimated the heart rate at rest conditions by applying a 10-s rolling window with an overlap of 50%. They also investigated the heart rate with motion disturbance by compensating the motion effects with the accelerometer measurement. The heart rate with motion was estimated by implementing a 20-s moving window with an 80% overlap. Both estimations were executed by using the red and infrared signals. The signals are detrended and added together before implementing the sixth-order FIR filter [40].

Signal processing techniques such as empirical mode decomposition (EMD), multi Gaussian decomposition (MGD), and pulse wave decomposition (PWD), have been utilized by several studies to decompose an in-ear PPG pulse wave into its individual components [18,42,45]. EMD employs a nonlinear bandpass filter array that decomposes the waveform into multiple amplitude and frequency components called intrinsic mode function (IMF). MGD decomposes the pulse wave into a sum of Gaussian-shaped waves with different amplitudes, widths, and locations. While PWD decomposes the in-ear PPG pulse wave into a sum of waves representing the different origins of the cardiovascular system.

Our literature search has revealed that another approach to PPG pre-processing involves the deconstruction of the time-domain signal into several amplitude and frequency (time series) components [18]. EMD removes noise from each IMF and recombines them to produce the pre-processed PP signal. The IMF of the PPG signal is defined prior to any noise component removal. This is accomplished by excluding the IMG at a specific frequency and then recombining the signal without the noise to produce the final pre-processed PPG signal.

The EMD algorithm can be used in multiple channels where each channel conveys variants of the same signal [18]. The multivariate empirical mode decomposition (MEMD) algorithm, generally used for EEG signals, has also been applied to PPG signals. The addition of independent noise channels to the MEMD algorithm has been shown to improve the extraction of IMF by enhancing frequency localisation and reducing the mode mixing between the IMFs. This method is known as noise-assisted multivariate empirical mode decomposition (NA-MEMD) [18]. Davies et al. added five white Gaussian noise channels to the raw PPG signals using the NA-MEMD to produce IMFs for each of respiratory modes, and then reconstructed the respiratory waveform using these IMFs [18].

MGD is used to decompose the signal into several Gaussian components to analyze the arterial pulse wave. This method assumed that the pulse wave constitutes the summation of several Gaussian distributions with different amplitudes, widths, and locations. It is useful to analyze the morphology of the signal. Each component has clear physiological and hemodynamic parameters [45].

Tigges proposed the PDA model, which represents a single pulse wave that is represented as a linear composition of basis functions, reflecting a pressure wave from different parts of the cardiovascular system where the first pulse can be attributed to ventricular contraction, while the second wave is believed to represent a reflection at the intersection of the thoracic and abdominal aorta given the considerable reduction in artery diameter. The third pulse wave is thought to be caused by a diameter change at the junction of the abdominal aorta and the iliac arteries [42].

#### 3.4.3. Feature Extraction

Some features can be extracted from the raw PPG signals without being pre-processed or filtered. Respiration-induced intensity variation (RIIV) can be observed from the raw PPG signal. The DC component of the PPG signal is modulated by changes in venous pressure, which gives rise to a signal related to respiratory activity. The respiratory frequency of the signal can be estimated by analyzing the normalized power spectral density of the raw PPG signal [18]. Davies et al. discovered that the PPG signal must be in an ideal situation with minimal movement over long time periods of 100 s for a better frequency estimation [18].

- Waveform features

Waveform features can be extracted directly form the PPG waveform. Among PPG features that has been studied in the in-ear PPG signal are pulse amplitude, pulse width and pulse rate. Davies et al. extracted three pulse amplitude variation and pulse interval variation from the in-ear PPG signal [18]. Pulse amplitude variations can be acquired from the AC filtered PPG signal’s envelope. This feature observed the changes in left ventricular stroke volume. Pulse interval variations in other hand can be determined by calculating the time span between successive pulses, and this feature was generated through respiratory sinus arrhythmia. It is observed that the spectral power of RIIV and pulse amplitude variation from the in-ear PPG signal was higher than the finger PPG signal [18]. 

Pulse rate can be extracted by detecting peaks and troughs. Our literature search found that the most frequently used peak and valley detection method for in-ear PPG is based on the maximum or minimum detection method (LM), window method (WM), gradient-based technique (GT) and machine learning. 

LM detection method is an approach to defining a maximum or minimum value within a specific area based on a predetermined threshold. The threshold can be either a fixed value or adapted to the specific region. Some studies utilized the LM method repeatedly in a moving window with a threshold adapted to each window. The window threshold coupled with the LM method finds the maximum value in data collected within a certain interval and window gap. The gradient-based method positions a point at which the slope becomes zero at the maximum value. This is widely used in Matlab under the “findpeaks” function. Davies et al. implemented a gradient-based method to find the local minima and local maxima of a signal by first identifying the slope direction of the signal [35,37]. Venema et al. detected the peak using the zero-crossing method where a threshold was defined at the point where the gradient of the pulse wave was at the maximum [43].

Vogel et al. explored the LM method by splitting the signals into smaller windows [20]. A fixed threshold was defined by computing the median and the standard deviation of the signal. Time intervals were defined at every pair of split points where a peak parallel to the *x*-axis was identified by the local maximum [20]. The time difference between two consecutive peaks divided by sampling interval is described as beat-to-beat delays. The average heart rate is determined using the median beat-to-beat delays.

Ferlini et al. extracted the respiration peak using the sliding window approach [14]. The IR signal was first filtered to eliminate the non-respiratory frequencies. A sliding window of 30 s was used to detect the peaks and compute the breaths per minute within each window. They also extracted HR and HRV from the peaks corresponding to heartbeats. The method used a local maximum detection method where all peaks representing the heartbeats were detected.

Pedrana et al. proposed an approach using a sliding window of 10 s where the filtered signal was inverted [40]. They set an adaptive threshold based on the pulse amplitude standard deviation and the minimum peak distance was fixed to 0.3 s considering the maximum heart rate in rest condition is 200 bpm. This approach minimizes errors on peaks detected by acknowledging the peak heights and distances.

- Physiological Features

Photoplethysmography has emerged as an important component in monitoring physiological features. There is a considerable amount of literature that has been published on observing clinical features including respiration rate, heart rate (HR), blood oxygen saturation, cardiac output, heart rate variability (HRV), thermoregulation, and vascular assessment [48]. Studies for detecting various clinical parameters have equally been conducted using the in-ear PPG [14,15,19,20,21].

Ferlini et al. extracted heart rate and heart rate variability from an infrared PPG signal compared to R-peaks amplitudes of an ECG signal [14]. The HR and HRV were extracted from the peaks that corresponded to the heartbeats where HR is the average number of pulses in a session and HRV is the mean time between consecutive pulses in a session.

They have also demonstrated the extraction of SPO2 and respiration rate features in the study. The SPO2 was calculated in a conventional way by extracting data from red and infrared PPG. In this method, the AC and DC components of the red and IR PPG signals are first separated, and then the ratio of the AC and DC components of each signal is computed. The ratio of the ratios of the red and IR signal is calculated to be used in the SPO2 calculation. SPO2 is computed with the following formula: SPO2 = A − BR, where A and B are calibration coefficients provided by the manufacturer and R is the ratio of the ratios of the red and IR signal. The respiration rate was extracted using IR PPG, which involved applying a bandpass filter to the signal between 0.1 and 0.8 Hz to eliminate the non-respiratory frequencies. The filtered signal was segmented into a smaller window to find peaks of respiration, using the average number of breaths per minute as the measure of the respiratory rate.

Vogel et al. presented a study that measured heart rate and heart activity based on in-ear PPG [17,20]. They developed two algorithms to determine the heart rate. The first algorithm utilized peak detection to calculate the heart rate, which has been described in section [17,20]. The second algorithm is known as dominant frequency detection (DFD), where the frequency spectrum of the signal was used to calculate the heart rate. The algorithm transformed the signal into the frequency domain using the fast Fourier transform (FFT), and a fourth-order Blackman–Harris window and zero-padding were applied to cut down spectral leakage and improve computation performance [20].

Davies et al. extracted three types of respiratory modes from the in-ear PPG signal, namely, respiration-induced intensity variations (RIIV), pulse amplitude variation, and pulse interval variation [18]. Pulse amplitude variations can be acquired from the AC-filtered PPG signal’s envelope. This feature observed the changes in left ventricular stroke volume. Pulse interval variations, on the other hand, can be determined by calculating the time span between successive pulses, and this feature was generated through respiratory sinus arrhythmia. It is observed that the spectral power of RIIV and pulse amplitude variation from the in-ear PPG signal was higher than the finger PPG signal. It is also reported that they extracted features based on the chronic obstructive pulmonary disease (COPD) waveform that includes features such as skewness, duty cycle, max, min, normalized max, and normalized min. The features would be used to classify COPD with healthy patients and other respiratory diseases. In leave-one-segment-out cross-validation, a random forest classifier was able to differentiate between COPD and non-COPD with an average accuracy of 92% and a specificity of 87% [18].

Venema et al. studied cardiorespiratory monitoring and heart insufficiency from the in-ear PPG [21,38]. A hypoxia study was first executed with arterial oxygen saturation monitors at several locations. Arterial oxygen saturation was computed with an R-curve, which was calculated with the detection of maximum and minimum values of each pulse [21].

The hypoxia study was done further with the in-ear PPG sensor coupled with a polysomnographic measurement system (PSG) for reference of the heart and breathing rate [38]. It extracted the signal amplitude variation (SAV) and cardiorespiratory coupling to estimate the breathing cycle. Using binary Naïve Bayes’ classifiers, it is possible to approximate the moment of inspiration and expiration with a mean sensitivity of 81.4% and mean specificity of 86%. This study has shown that there is the possibility to measure simultaneously breathing rate and heart rate with an in-ear PPG sensor.

The study also defined two classification parameters to separate people suffering from heart insufficiency, which include the normalized standard deviation of the pulsatile component (calculated as the standard deviation of the pulsatile component divided by its mean value) and the variability of heartbeats (measured by the standard deviation of consecutive beat-to-beat intervals). The pulsatile component was used in assessing the cardiac activity as the arrhythmia resulted in a decrease in the pulsatile component (AC) of the PPG signal coupled with high fluctuation in frequency. Based on the classification performed by Venema et al., an automatic classification between heart failure and a normal cardiac rhythm can be executed.

Venema et al. investigated cardiology and respiratory monitoring during sleep [43]. They computed the heart rate with a fourth-order Butterworth filter as mentioned previously. The standard equation was utilized to determine the arterial oxygen saturation, where the ratio of the ratio between the red and infrared PPG signal was computed. This allowed for the computational of SPO2 to identify sleep apnea among subjects. The study explored three distinct methods of respiration detection, which include respiration rate calculated by segmenting PPG amplitude, segmenting SPO2 variation, and using the respiratory sinus arrhythmia (RSA) [43]. The RSA is described as the effect of an increase and decrease in heart rate due to inspiration and expiration, respectively [43]. The RSA was computed in three stages: first, the calculation of the heart rate using the zero-crossing method; second, a second-order Butterworth filter with cut-off frequencies of 0.1 and 0.33 Hz was applied to the signal; and third, a zero-crossing detection and calculation of subsequent distances and interpolation. However, the RSA depended on various parameters and the method explained in the paper provided no reliable information on the breathing rate.

Pedrana et al. carried out an investigation into the heart rate estimation in rest conditions and in motion [40]. In both conditions, a moving difference filter was applied. The PPG signal was filtered in the FIR differentiator filter with a sliding window of 10 s with an overlap of 50%. The PPG waveform was sampled at 100 Hz. The peaks were detected with a peak detection algorithm after inverting the filtered signal. The heart rate was determined by calculating the mean period between two successive peaks. To validate the heart rate algorithm during rest, the mean absolute error (MAE) was computed. The algorithm exhibited an average error of 1.042 bpm [40].

In analyzing the heart rate with motion compensation, an accelerometer was mounted to estimate the movement. The accelerometer signal was filtered based on the adaptive filtering method with a rolling window of 20 s with an overlap of 80%. The PPG waveforms were sampled at 500 Hz and processed similarly to the previously described paragraph. The acceleration magnitude was processed by applying a fourth-order Butterworth band pass filter with a cut-off frequency ranging from 0.5 Hz to 10 Hz. Discrete Fourier Transforms (DFTs) were performed on both the filtered PPG signal and the acceleration magnitude. If any motion disturbance was detected, a second order of IIR notch filter was conducted in the time domain for the PPG signal. The heart rate was then estimated by calculating the DFT of the filtered signal and selecting the dominant frequency as the estimated heart rate. The average error of the algorithm was found to be 2.77 bpm [40].

- Motion Artifacts

The nature of a wireless earbud is that people can wear it on the go. Artifacts caused by movement can influence the PPG signal quality. For in-ear PPG devices, jaw movement has the strongest influence on the signal [20]. Vogel et al. addressed the question of motion in the in-ear PPG signal and found the two principal causes of motion are walking and jaw movement [39]. They discovered that walking has almost no influence on in-ear PPG data, but jaw movements increase the artifact amplitude of the in-ear PPG signal. Motion artifacts can be differentiated from the in-ear PPG signals when the pulse contour can be clearly visible. Vogel et al. indicated that the step frequency could influence the walking artifacts. This study is not able to distinguish pulse wave and chewing artifacts when the artifacts’ frequency dominates the PPG signal, and it was not able to differentiate the signal from the walking artifacts when the range of heart rate and the artifacts’ frequency were almost similar [39].

An experimental demonstration of the effect of jaw movement on the PPG signal was carried out by Passler et al. by analyzing the signal of chewing gum and talking during data recording. Passler et al. set up the experiment using a Cossinus One and Dash Pro in-ear PPG device to capture the PPG data. It was reported that the signal disturbance due to jaw movement was too severe to determine an accurate waveform of the pulse rate [39]. They also observed that movement due to a body posture change and fixing the sensor can also result in intense noise. These data were not considered in the statistical analysis of the study.

Ferlini et al. set up an experiment on two devices to collect a PPG signal from different sites of the ear and examined motion activities including speaking, walking, and running [14]. This was to explore the effects of movements for the different positions of the PPG sensor. They concluded that positioning the PPG sensor in the ear canal has less impact on the signal. HR, HRV, and SPO2 can be observed from inside the ear canal with a lower median error. However, a higher error was reported when they calculated the respiration rate.

Davies et al. analyzed the in-ear respiratory waveform with and without motion. They concluded that motion artifacts corrupt the features of PPG waveforms. This caused them to eliminate the signal with motion artifacts in their feature calculation as it was believed to reduce accuracy in the COPD prediction [18].

Among all research on in-ear PPG, Poh et al. and Pedrana et al. have investigated adaptive noise cancellation to deal with motion artifacts [40,49]. The PPG sensor output is believed to be a combination of the physiological signal of blood volume and motion artifacts. The motion-induced noise is estimated by processing the accelerometer signal where it is then subtracted from the corrupted PPG signal through an adaptive filter.

Pedrana et al. explored the heart rate estimation algorithm with and without motion [40]. The current study utilized an accelerometer installed at the in-ear PPG board to eliminate the motion of the PPG signals. A second order of IIR notch filter was conducted in the time domain to the PPG signal if motion disturbance was detected. This is because the motion artifacts can reduce the quality of the signal and amplify the frequency components of the PPG signal. The DFT of the PPG signals after the notch filter application was calculated to estimate the heart rate. In this study, the average error of the heart rate with motion compensation was equal to 2.77 bpm when compared with the reference value acquired from the ECG.

Vogel et al. developed an in-ear PPG sensor for long-term monitoring, which is called In-MONIT [17,20,50]. The system estimated heart rate variability in a motion artifacts signal by eliminating the signal and estimating the missing data by calculating Lomb-periodograms.

## 4. Discussion

### 4.1. In Ear PPG Principle and Placement

Based on previous studies, they have shown that the PPG signal obtained from the ears are more accurate and reliable than finger PPG in conditions such as hypothermia, hypovolemia or sepsis [15,16,42,51]. During these conditions, peripheral perfusion may be compromised which led to reduce signal quality in finger PPG. The ear PPG signal remains unaffected as the ear canals are believed to be unaffected by cardiovascular centralization. Furthermore, ear PPG is considered more accurate because of the higher density of blood vessels compared to the finger. This led to stronger signal as PPG technique measures the variations of blood volume. The higher vascularity improved accuracy and the signal has better quality. 

In-ear PPG has also demonstrated a faster response to changes in blood oxygen levels compared to finger PPG [16,51]. The faster response to blood oxygen level changes provided by in-ear PPG is highly valuable in multiple conditions. It enhances the effectiveness of detecting medical conditions such as hypoxia, sleep apnea and real-time monitoring of high-intensity activities. In-ear PPG’s ability to promptly respond to blood oxygen saturation changes allows for immediate feedback, empowering individuals to adjust their activity levels and mitigate potential complications. 

Early detection of blood oxygen saturation changes particularly important in the cases of hypoxia and sleep apnea. These conditions require early medical intervention to prevent potential complications and minimize the risks associated with inadequate oxygen supply. Furthermore, during exercise and high-intensity activities, the capability of in-ear PPG to provide rapid response allows for immediate feedback on blood oxygen saturation changes, enabling individuals to adjust their activity levels accordingly and prevent from any adverse outcomes. 

**Figure 5:**
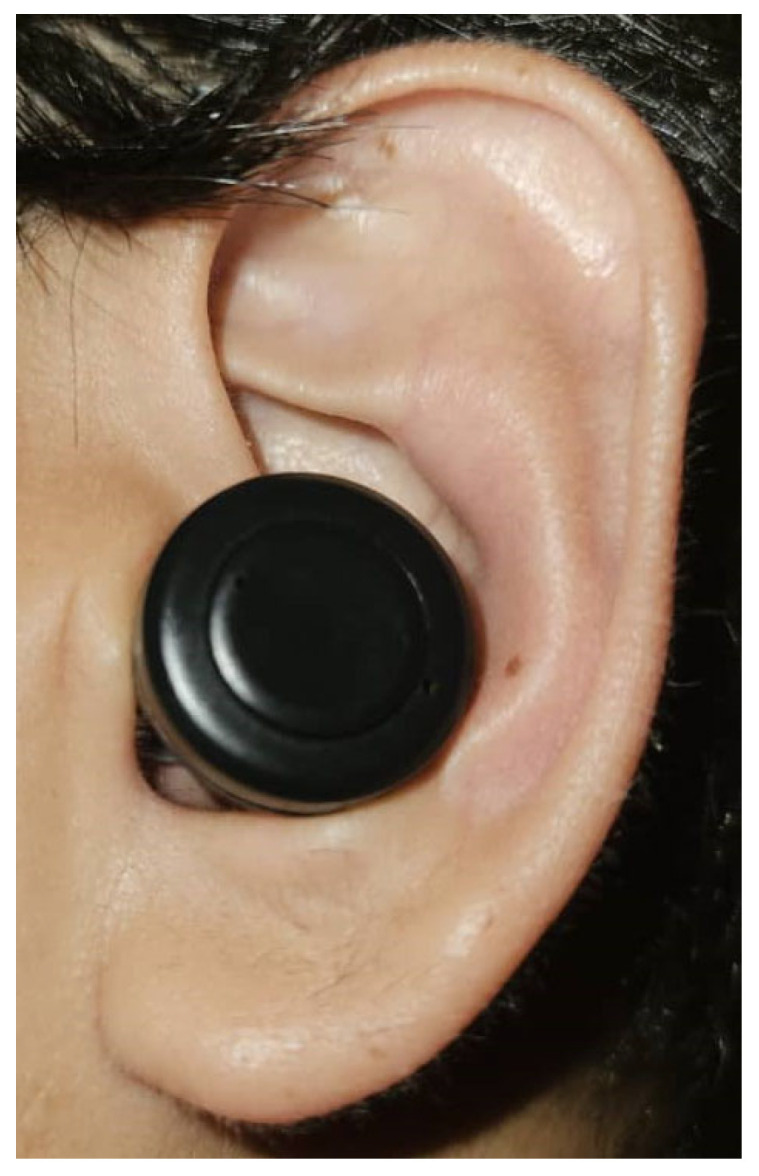
Example of PPG placement inside the ear canal.

The position of the in-ear PPG offers an added advantage. The discreet location of the sensor inside ear canals which is partially hidden from the view of others (Figure 5). The adoption of earbuds for telecommunication has been widely accepted. Incorporating healthcare monitoring features within the earbud enables a smooth integration of the device into daily lives, without the need for external and visible devices that may attract attention. The position of the sensor inside the ear canal reduces interference from external factors [20,51]. The ear is located in a more controlled environment compared to the finger, which is exposed to temperature changes and ambient light. The ear is also less susceptible to movement artifacts compared to the finger. It remains relatively stable and is less influenced by body movement. However, it is important to note that movements of the mouth such as talking and eating can impact the ear PPG signal. In contrast, the finger is more prone to movements, which increases the risk of motion artifacts that can affect the PPG signal. 

**Figure 6:**
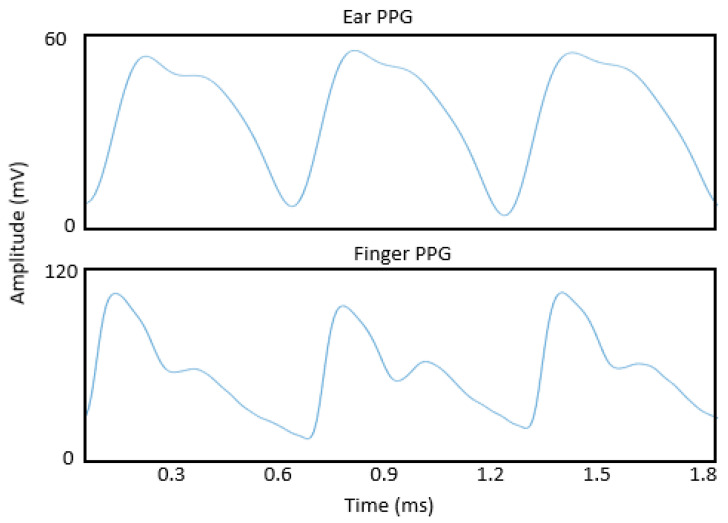
Difference in morphological signal between in-ear PPG and finger PPG.

The morphology of in-ear PPG waveform differs from those acquired from the finger. Dicrotic notch, one of PPG pulse features during diastolic phase is distinctly noticeable in the signal obtained by finger PPG compared to the ear canal PPG signal. The dicrotic notch in ear PPG signal arrives faster compared to the finger PPG signals [51] (Figure 6). The differences in the PPG pulse morphology may be due to the changes with vascular tone and compliance [31,32,33,34]. Budidha also mentioned that the PPG signal obtained from the ear canal exhibits a strong resemblance to the arterial pressure pulse recorded from the aortic arch, where cardiovascular function information can be extracted by performing further decomposition of the in-ear PPG signal [51]. Limited knowledge exists regarding the morphological variations between in-ear PPG signal and other parts of the body such as the finger PPG. Currently, only Budidha has conducted a study on the difference in morphology of the PPG signal from the ear canal. Further research is required to explore and understand the differences comprehensively. 

### 4.2. In-Ear PPG System

The in-ear PPG system comprises a photodiode and LED positioned at one side of the sensor. Previous studies related to in-ear PPG primarily employed the reflectance mode, where the light on the skin was reflected and captured by the photodetector located next to it. The transmission mode was only applied in studies where the sensor was placed on the earlobes. To prevent optical crosstalk that can reduce signal accuracy, an optical barrier was positioned between the receiver and the detector. All previous studies utilized three common LED colours, which are green, red, and infrared. However, most studies used red and infrared LED instead of green LED (see Table 3), which is possibly due to power consumption optimization. The IR LED consumes less power for the same amount of output compared to the green LED, and green radiation is absorbed more by melanin which is not suitable for darker skin [20,40]. Vogel et al. found that the infrared signal has better signal noise ratio compared to red LED which is different from the results presented by Budidha et al. which shows that both red and infrared PPG can acquire good quality of PPG signal from the ear canal. The difference of findings may be caused by the different of position in the ear canal, wavelength and sensors used by both authors. 

### 4.3. In-Ear PPG Analysis

Previous studies have shown that in-ear PPG has an advantage in the placement of a wearable. The location of the ear, which is nearer to the heart, gives a faster response to blood oxygen changes compared to the finger PPG, and it can accurately detect hypoxia and sleep apnea [19,21]. In-ear PPG has also proven to be resistant to environmental changes such as temperature and light as the PPG sensor will be inserted into the ears and removes the surrounding noise. Little is known about in-ear PPG signal quality, and it is not clear if the PPG signal from the ear is better than PPG signal from the finger.

Most studies on in-ear PPG signal processing have only focused on frequency filtering. Frequency filtering is a popular technique for retaining only the main frequency that is useful in PPG signals. It is an advantageous method in removing the range of noise that is not part of the physiological signal which overlaps the PPG signal. Still, frequency filtering is limited to stationary noise. The use of in-ear PPG is expected to be in an environment with a lot of movements. Hence, non-stationary noise from motion artifacts should be handled effectively.

While most research has relied on frequency filtering, only one study has attempted to investigate time-frequency filtering where the study processed the in-ear PPG using EMD. Time-frequency filtering such as wavelet-based processing and EMD are alternative methods in processing the PPG signal to deal with a non-stationary noise frequency that overlaps with the PPG signal. PWD is another option for processing in-ear PPG signals. PWD with a higher number of kernels has shown to be reliable and robust in processing in-ear PPG with noise and motion artifacts. Further research is necessary to determine the optimal usage of various types of signal decomposition. Additional studies are required to investigate each different physiological application of in-ear PPG.

The challenges in processing the in-ear PPG signals include baseline noise, motion artifacts, and low amplitude of the PPG signal. Baseline wander is a low-frequency noise on a PPG signal that can be removed efficiently by the frequency filter as its spectral content is usually situated below 1 Hz in rest conditions; higher frequencies may be expected during walking or exercise. A substitute technique other than frequency filtering is required to remove the noise conclusively. Examining a corrupted signal before further processing the signal is recommended. A previously developed best pulse selection algorithm can be introduced to validate a good signal [52].

The low amplitude of a PPG signal is another limitation in processing an in-ear PPG signal. The PPG waveform can cause abrupt amplitude variations due to the automated gain controller that relies on the amplitude of the input signal [31]. Additionally, poor contact between the sensor and the ear can also result in low PPG signal amplitude. Furthermore, low perfusion of the PPG signal can also indicate a loss of central blood pressure and vasoconstriction in the tissue, which is unlikely to be observed from the in-ear PPG. The low PPG signal amplitude poses difficulties in detecting heartbeats and extracting features. Therefore, improving the in-ear PPG signal amplitude and quality is needed. Further research in low perfusion signals is required to completely eliminate the distortion and noise of PPG signals.

Motion artifacts resulted in movement and vibrations of the sensor, which led to poor contact between the sensor and the tissue. Even the slightest change of body position and the adjustment of the sensors can cause a strong signal noise. It is important to identify the motion artifact and process the signal precisely, especially with excessive movement that can distort the signal. The consequence of severe motion artifacts can impact the waveform and the signal and can corrupt the calculation for features extraction. Hence, any motion artifacts must be eliminated or fixed before further analysis.

Motion artifacts caused by the movement of the jaw are the most common sources of errors in in-ear PPG measurements. Activities such as speaking and eating have a greater influence on the signal compared to walking. However, the ear canal has been found to be less affected by other movements, making it a favourable position for PPG sensors.

ANC has been proven effective in heart rate measurements, as demonstrated by Pedrana et al. and Poh et al. [40,49]. This method involves the acceleration measurement using an accelerometer, which is then adapted to the original signal. However, most studies have utilized only the vertical axis of the accelerometer, ignoring the other two axes. It is important to study the potential of multi-axis accelerometer signals as it would help to mitigate the effects of sensor misalignment during activities and increase the accuracy of the signal.

Another unique approach by Wartzek et al. analyzes in-ear PPG recording with motion artifacts; they study how much data loss affects the signal. However, this method is not suitable for real-time long-term monitoring [50].

### 4.4. In-Ear PPG Applications

Much of the previous research on in-ear PPG applications has focused on exploring the technology for health monitoring especially to monitor respiratory and cardiovascular systems. Various studies have assessed the use of pulse oximetric sensors in the ear. What we know about measuring blood oxygen saturation is largely based on laboratory settings. Several research suggest that the measurement of SPO2 using in-ear PPG is more accurate in several conditions such as hypothermia, hypovolemia, and sepsis [15,16,42,51]. This is due to peripheral perfusion and cardiovascular centralization having minimal effects on in-ear PPG signals. These findings may be somewhat limited by laboratory conditions. It is interesting to compare the blood oxygen saturation from in-ear PPG in various conditions, with motion artifacts, and in clinical settings.

Preliminary work on investigating in-ear pulse oximetric sensors in clinical settings was undertaken by Venema et al. [13]. They investigated blood oxygen saturation during different surgeries where the PPG sensors were put inside the ear. However, the study reported issues with the measurement specificity for pulse oximetry. More research on in-ear PPG focused on real clinical settings and actual conditions needs to be done as the real environment is more challenging than laboratory settings with multiple electromagnetic devices and different radiations in the surroundings.

Several studies explored heart monitoring using the in-ear PPG. Most studies in this group focus on heart rate and heart rate variability measurement. HR and HRV can be achieved easily by identifying the peaks of the pulse wave. More complicated measurements such as stroke volume and cardiac output that have been measured by other PPG such as wrist and finger PPG have not been investigated by any research groups that study in-ear PPG.

More recently, blood pressure monitoring with in-ear PPG technology has been investigated. Although most research used in-ear PPG as a secondary signal together with ECG [53], Xing et al. estimate the blood pressure using only in-ear PPG. Multi Gaussian decomposition and pulse wave decomposition have been used to study the hemodynamic of the arteries, which can reflect the blood pressure measurement [42,45]. Each decomposition has a physiological meaning. The BP estimation used analytical features from PPG waveforms and their derivatives, time-domain features, frequency-domain features, and statistical features.

**Figure 7:**
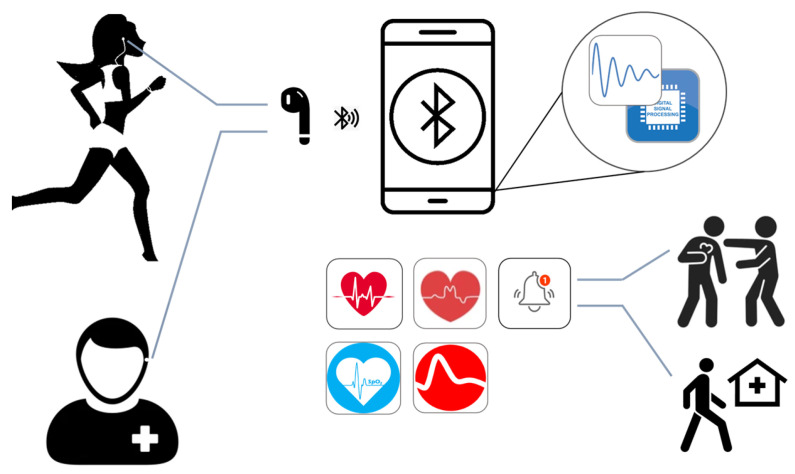
The possible application of in-ear PPG in remote health monitoring and fitness tracking.

The use of ear PPG is promising in some notable applications mainly in remote monitoring and fitness tracking. In-ear PPG can provide insight into patient’s continuous vital measurements, enables the possibility of remote monitoring, allowing healthcare professionals or family members to monitor patient’s health condition remotely. By establishing a seamless integration of in-ear PPG with mobile devices through wireless connections such as Bluetooth, facilitates this capability (Figure 7). In-ear can detect vital signs during physical activities monitor exercise intensity and provide real-time feedback on heart rate and oxygen levels. The compact size and ability to be inserted into the ears offer promising technology for fitness monitoring. The convenience and portability of in-ear PPG make it an attractive option for individuals who desire continuous monitoring of their health conditions. 

Apart from remote and fitness monitoring, the paper has presented a wide range applications in cardiovascular and respiratory monitoring. In-ear PPG has great potential in these applications especially in monitoring vital signs and detecting different cardiovascular and respiratory conditions. In-ear PPG can effectively monitor heart rate, blood pressure, respiratory rate, respiratory efforts and identify cardiovascular and respiratory conditions such as sleep apnea, hypoxia, hypovolemia and arrhythmias which can be a valuable tool to be used at medical establishments such as hospitals and clinics. Further research is necessary to achieve optimal accuracy and specificity of in-ear PPG. In-ear PPG has the capacity to revolutionize continuous monitoring by providing comfortable, convenient and non-invasive. 

## 5. Conclusions

This paper provides an overview of the existing literature on in-ear photoplethysmography and its usage in physiological monitoring. In-ear PPG has gained popularity due to its advantage in the wearable position as earbuds. We have reviewed the current technology of the in-ear PPG including its principle, system, and processing technology and its applications in clinical and physiological monitoring. Furthermore, we have identified the potential limitation and challenges that in-ear PPG may face in processing the signal.

Recent advances in the in-ear PPG have demonstrated the potential of this technology to address clinical needs, particularly for long monitoring of cardiovascular and respiratory systems. However, to maximize the possibility of utilizing in-ear PPG signals and to gain a comprehensive understanding of in-ear PPG technology, there are several critical issues that require attention and further investigation. One of the issues is the need for validation studies against gold-standard measurements. It is crucial to validate the technology and verify its reliability. Another significant challenge is addressing the impact of motion and exercise on the in-ear PPG signal. Current available studies are very limited and require more investigation. Lastly, there is a gap in clinical application evaluations of in-ear PPG. While current research is conducted in laboratory settings with controlled conditions, it is essential to explore the impact of real-world environments on the accuracy of signals. This paper serves as a preliminary step for researchers to extend the future directions of in-ear PPG research.

## Figures and Tables

**Table 1:** Study of PPG placement.

Study	Sensor Position
Vogel et al., 2009 [20]	Near tragus and deeper inside the auditory canal
Poh et al., 2012 [12]	Tragus
Venema et al., 2012, 2013 [21,43]	PPG on tragus and measuring system behind the auricle
Venema et al., 2014 [13]	Inner part of tragus
Tigges et al., 2017 [42]	Ear canal (Sensors in two positions: radially oriented and axially oriented)
Budidha and Kyricou, 2018 [16]	Concha of the ear with an ear hook hang on top of the helix
Pedrana et al., 2020 [40]	Thumb (R/L), forefinger (R/L), middle finger (R/L), posterior wrist (R/L), anterior wrist (R/L), forehead, nasal bridge, ankle (R/L), ear canal (R/L), manubrium and xiphoid process (sternum area)
Ferlini et al., 2022 [14]	Behind the auricle, front of the auricle and the ear canal
Davies et al., 2022 [18]	Ear canal

**Table 2:** Pre-processing methods and details.

Pre-Processing Method	Details	Purpose
Frequency filtering	Linear FIR [16] Butterworth FIR [40] Bandpass and notch filter (0.5 Hz–10 Hz) [14] Bandpass 0.9–30 Hz [18,19]	Trend reduction Reduction for high-frequency noise
Empirical mode decomposition	Noise-assisted multivariate empirical mode decomposition (NA-MEMD) [18]	Low frequency reduction Noise reduction Baseline reduction
Multi Gaussian decomposition	Each pulse was decomposed into five Gaussian components: the two Gaussian function models the main forward pulse, the three Gaussian function represents different pulse reflections in the system [45]	Each component has a physiological meaning Noise reduction Highlight analytical features
Moving difference filter	Sixth-order FIR differentiator filter [40] Calculating the sample difference within a window size of a moving window	Eliminate high frequency noise Highlight PPG slope
		Noise reduction
Moving average filter	Calculating the sample average within a window size [21]	Noise reduction

**Table 3:** Comparison of ear PPG systems for physiological monitoring.

Features	Vogel et al. (2009) [17,20]	Pedrana et al. (2020) [40]	Ferlini et al. (2022) [14]	Poh et al. (2012) [12]	Poh et al. (2010) [49]	Passler et al. (2019) [39]	Xing et al. (2021) [45]	Venema et al. (2012, 2013) [13,21,43]	Davies et al. (2020)	Budidha et al. [16,51]
**Ear location**	Tragus near auditory canal	Ear canal	(1) Behind the ear, (2) Front of the auricle (3) Ear canal	Tragus	Ear Lobe	External auditory canal	Ear canal	Tragus	Ear canal	Ear canal
**Attachment to the ear**	Otoplastic insertion	Inserted inside the ear	Hook on the ear	Inserted inside the ear	Magnetic earring	Inserted inside the ear and hook	Inserted inside the ear	Inserted inside the ear	Inserted inside the ear	Inserted inside the ear
**Wireless/Bluetooth**	Yes	Bluetooth	NA	Bluetooth	Bluetooth	wireless	No	No	No	No
**Motion analysis**	No	Yes Adaptive noise cancellation	Yes	No	Yes Adaptive noise cancellation	No	No	No	No	No
**Physical testing**	Walking, jaw motion	Walking	Speaking, walking, running	Standing, walking, cycling, exercise	Walking, running	Cycling	No	Surgery in operational room	No	Cold exposure, ice water immersion
**Physiological measurement**	HR, HRV	HR	HR, HRV, SPO2, RR	HR	HR	HR	Blood pressure	HR, RR, SPO2, hypoxia	SPO2, COPD	SPO2, body temperature, hypothermia
**LED colours/wavelength**	Red and infrared	Green, red, and infrared	Infrared and red	Infrared	Infrared (940 nm)	Infrared and green	Green (550 nm)	Red and infrared	Green, red, and infrared	Infrared, red
**Accelerometer**	2D and 3D accelerometer fixed on head	3D accelerometer	None	No	3D accelerometer	No	No	3D accelerometer	No	No
**Reference**	Finger PPG, ECG	ECG	Finger PPG, ECG	ECG	ECG	ECG-Bodyguard 2	spygmomanometer	Finger PPG, ECG	Finger PPG	Laser Doppler flowmeter, tympanic thermometer, ECG
